# Novel approach to assess sarcopenia in children with inflammatory bowel disease

**DOI:** 10.3389/fped.2024.1204639

**Published:** 2024-11-19

**Authors:** Kriszta Katinka Boros, Gábor Veres, Hajnalka Krisztina Pintér, Éva Richter, Áron Cseh, Antal Dezsőfi, András Arató, George S. Reusz, Dóra Dohos, Katalin E. Müller, Orsolya Cseprekál

**Affiliations:** ^1^ Pediatric Center, MTA Center of Excellence, Semmelweis University, Budapest, Hungary; ^2^ Department of Internal Medicine, Pediatrics Clinic University of Debrecen, Clinical Center, ÁOK, DEKK, Debrecen, Hungary; ^3^ School of Ph.D. Studies, Semmelweis University, Budapest, Hungary; ^4^ Institute for Translational Medicine, Medical School, University of Pécs, Pécs, Hungary; ^5^ Department of Gastroenterology and Nephrology, Heim Pál National Pediatric Institute, Budapest, Hungary; ^6^ Department of Health Care Methodology, Faculty of Health Sciences, Semmelweis University, Budapest, Hungary; ^7^ Department of Surgery Transplantation and Gastroenterology, Semmelweis University, Budapest, Hungary

**Keywords:** fat-free mass (FFM), skeletal muscle mass (SMM), body fat mass (BFM), bioelectrical impedance analysis (BIA), inflammatory bowel disease (IBD), body composition (BC), body mass index (BMI)

## Abstract

**Introduction:**

Sarcopenia is associated with poor clinical outcomes in chronic diseases. Our study aimed to characterize body composition (BC) parameters in patients with inflammatory bowel disease (IBD) and compare skeletal muscle mass (SMM) parameters with the healthy pediatric population.

**Methods:**

BC of healthy controls (HC) and of patients with IBD were measured via multifrequency bioelectrical impedance (InBody 720 device) in a cross-sectional manner. The effect of sex, age, height, weight, and body mass index (BMI) on BC parameters, with a special attention to SMM, was assessed. Reference tables from SMM were generated using a maximum-likelihood curve-fitting technique for calculating *Z* scores.

**Results:**

BC parameters were associated with age, body size, and sex. SMM was lower in patients with IBD (*n* = 57, aged 6.71 ± 8.7 years) compared to unadjusted HC (*n* = 307, aged 9.9–19.3 years; 143 males; SMM: 22.34 ± 8.38 vs. 24.4 ± 6.3 kg; *p* = 0.03). SMM showed a moderately strong correlation with age, weight, height, and BMI (*R* = 0.65, 0.9, 0.87, and 0.66; *p* < 0.05 for each) in HC. In multivariate stepwise, ridge regression analysis, age, sex, and BMI remained the significant predictors of SMM (age β = 0.47, −0.31, and 0.38, respectively; *p* < 0.05). SMM of sex-, age-, and BMI-adjusted HC did not differ from IBD. Therefore, BMI *Z* score–based references were plotted for normalizing SMM, and SMM *Z* score was calculated and found to be similar to that of HC.

**Conclusions:**

BC is supposed to be an easy-to-measure and objective marker of sarcopenia in children with IBD. Adjustment of SMM for BMI *Z* score might be needed to avoid the overestimation of sarcopenia in this patient population.

## 1 Introduction

Sarcopenia or loss of muscle mass is a common phenomenon of malnutrition in patients with chronic diseases (1). The decreased nutrient intake, lack of physical activity, and the large amounts of inflammatory cytokines lead to a catabolic state with degradation of muscle mass, turning into significant muscle loss (2). In adults, sarcopenia is associated with declining functional performance, falls, and overall mortality. Pediatric aspects of sarcopenia have only been discussed in recent years, reporting that it is associated with clinical outcomes such as extended hospitalization and increased morbidity and mortality in children with chronic diseases (3, 4). Moreover, pediatric sarcopenia may have negative effects on growth, neurodevelopment, and fine motor development and impair the quality of life (3, 5). In adults, sarcopenia has been defined by the loss of skeletal muscle mass (SMM) and reduced muscle strength or physical performance (6). The consensus definition of sarcopenia and reference values of SMM and other body composition (BC) values in children are yet to be defined (4).

Inflammatory bowel disease (IBD) is a chronic, incurable disease of the gastrointestinal tract. Diet (suboptimal energy intake, malabsorption) and disease severity (enteric nutrient loss, increased basal energy expenditure, used medications) are key factors influencing nutritional status and also BC (7). In patients with IBD, sarcopenia can be a predictor for adverse clinical outcomes and can increase the risk of postoperative complications (8, 9). Moreover, according to the BE-FIT-IBD studies, conducted in adults, patients with IBD show a lower physical activity, regardless of sex, disease subtype [Crohn's disease (CD) or ulcerative colitis (UC)], and disease activity. Based on this study, the fear of engaging in physical activity stems from the environment and social networks in which they grew up, leading to an endangerment of developing sarcopenia (10, 11). Evaluation of BC to identify patients who are at risk of sarcopenia might contribute to the optimization of nutrition and medical treatment to prevent subsequent complications, such as the increased risk of requiring surgery, with the high rate of major postoperative complications, abscesses, longer hospital stay, and increased risk of infections (12).

There is wide heterogeneity in BC methodologies used to measure SMM. Different methodologies measure different parameters, describing muscle mass: computerized tomography (CT) scan and magnetic resonance imaging (MRI) can measure the psoas muscle area (PMA), peripheral quantitative computed tomography (pQCT) shows the cross-sectional muscle area, and the dual-energy x-ray absorptiometry (DXA) calculates muscle mass. One of the most recommended measurement techniques is bioelectrical impedance analysis (BIA) due to its cost-effectiveness, reproducibility, and easy-to-use approach (13). Because of the different methodologies and the differently measured BC parameters, cut-off values for describing sarcopenia have yet to be provided and unified.

Despite the fact that correct diagnosis and careful follow-up of sarcopenia might help identify high-risk populations among those having chronic wasting diseases, there is still a lack of evidence on which approach should be used to diagnose malnutrition in children at high risk (4). Furthermore, threshold specification for pathological muscle mass loss is still needed due to the missing reference database for the healthy controls (HC). In previous studies evaluating sarcopenia in children, some authors compared SMM to age- and sex-matched HC; some used previously published age- and sex-specific SMM reference parameters; and some defined sarcopenia as the lowest tertile or quartile or less than −1 to −2 standard deviation of muscle fat ratio (8, 14–19). However, there is still no consensus whether SMM is the best choice to monitor high-risk pediatric patients with IBD.

In children, all structural and functional parameters change dynamically during growth. Peripheral and central blood pressure, central pulse wave velocity, or body mass index (BMI) depends on age and also differs according to sex (20–22). Therefore, the determination of childhood percentile values for these parameters has become mandatory in our everyday practice. The concept of considering a parameter as abnormal is defined as a deviation from the population average adjusted for sex, age, and body size. This view of evidence has been accepted worldwide for decades. As far as the characteristics of BC parameters and their association with age, sex, and their relation to the growing process are concerned, reference values for BIA parameters have not been studied so far. Therefore, our aim was to assess if muscle loss measured by SMM is represented in the IBD group as compared to the healthy population and to scrutinize the associating factors of different BC parameters in healthy children.

## 2 Patients and methods

### 2.1 Subjects

In our single-center observational study, patients with IBD were consecutively recruited between September 2016 and April 2018 at the 1st Department of Pediatrics, Semmelweis University, Budapest, Hungary. Patients with newly diagnosed IBD or starting anti-tumor necrosis factor (TNF) therapy (due to insufficient response to conservative therapy), aged between 10 and 18 years, were asked to participate in the study. Exclusion criteria were concomitant conditions affecting BC [e.g., edema, hypoalbuminemia, cirrhosis, associated endocrine or chronic disorders, known active malignancy, or fracture (especially leg fracture)]. All participants were of Caucasian descent. IBD was diagnosed on the basis of the Porto criteria, and patients were treated according to the ESPGHAN guidelines (23–25). Disease activity was determined by the Pediatric Crohn's disease Activity Index (PCDAI) and Pediatric Ulcerative Colitis Activity Index (PUCAI) scores (26, 27). Further disease-specific information (e.g., disease location, laboratory parameters) was obtained from the medical records of the patients stored in the official clinical software.

BC analysis of the HC was performed in the frame of our previous cohort study aimed at measuring pulse wave velocity in the healthy population (28) (OTKA 071730 National Scientific Research Fund, participating authors: GR and OC). All children had parental informed consent and were examined at local primary and secondary schools between 2007 and 2012. Inclusion criteria were age between 10 and 18 years and absence of known chronic or acute disease. Exclusion criteria were hypertension (systolic and diastolic blood pressure below 90th percentile according to age, gender, and BMI), any cardiovascular disease, or obesity (BMI percentile below 95 and above 5) (29).

### 2.2 Methods

Characteristics of the patients with IBD, anthropometric parameters, and routine laboratory values were recorded at the time of BC measurement. Height and weight were measured by trained staff by using validated fixed stadiometers, at the time of BC measurements, with the same clothing and conditions (see below). BMI *Z* score values were calculated at baseline (Table 1 and Supplementary Table S2).

**Table 1:** Baseline anthropometric data of unadjusted healthy controls and children with IBD.

Variables	Controls		Patients	
	Mean	95% CI	Mean	95% CI
*n*	307		57	
Age (years)	14.3	14.0 to 14.5	14.2	13.5 to 15.0
Sex [males (%)]	143 (47)		32 (56)	
Height (cm)	164.2	162.9 to 165.6	161.9	158.1 to 165.8
Height *Z* score	0.4	0.3 to 0.6	0.3	0.0 to 0.6
Weight (kg)	53.2	51.8 to 54.6	46.9*	43.7 to 50.1
Weight *Z* score	0.04	−0.1 to 0.1	−0.5*	−0.8 to −0.2
BMI (kg/m^2^)	19.5	19.2 to 19.8	17.6*	19.6 to 20.4
BMI *Z* score	−0.2	−0.3 to −0.1	−0.7*	−0.9 to −0.5
SMM (kg)	24.4	23.7 to 25.1	22.3*	20.1 to 24.6
FFM (kg)	43.7	42.6 to 45.0	37.9*	35.4 to 40.6
TBW (L)	32.1	31.3 to 32.9	27.9*	25.9 to 29.8
BFM (kg)	9.5	8.8 to 10.2	8.6	7.2 to 10.0

CI, confidence interval; BMI, body mass index; TBW, total body water; FFM, fat-free mass; BFM, body fat mass; SMM, skeletal muscle mass.

* *p* < 0.05.

BC was measured using the same multifrequency (MF) bioelectrical impedance equipment (InBody 720; Biospace Co, Ltd, Seoul, South Korea) both in HC and IBD groups (29). The technical validation of the applied method and the device were confirmed previously in adults and children as well (30–33).

InBody 720 measures segmental impedances at the four limbs and trunk altogether with eight tactile electrodes by 250 mA alternating electrical current at multiple frequencies of 1, 5, 50, 250, 500 and 1,000 kHz. The electrodes and sensors measure segmental impedances from each extremities and the trunk. The total body impedance value is calculated by summing the segmental impedance values, which directly provides information about total body water (TBW), body fat mass (BFM), fat-free mass (FFM) and SMM. Measurements were performed before noon (8.00–12.00) and after at least 2 h of fasting in minimal clothing, without shoes, with abducted upper (30°) extremities without jewelry and watches.

### 2.3 Data analysis and statistics

Data analysis was performed by using the STATISTICA 8.0 (StatSoft Inc.). Data have been presented as mean and 95% confidence interval, unless indicated otherwise. Normal distribution of data was determined with the Shapiro–Wilk test and normal probability plot analysis. Height, weight, and BMI values were converted to age- and sex-specific standard deviation *Z* scores based on Hungarian standard reference charts from Joubert et al. (34). Anthropometric, laboratory parameters and other data with normal distribution were compared with Student's *t* test or ANOVA where appropriate. Variance analysis of data with non-normal distribution was performed with the Mann–Whitney *U* test.

The associations between anthropometric parameters and BC parameters were examined by means of linear univariate and multivariate stepwise ridge regression models.

Once BC parameters of patients with IBD were compared with the entire HC group, anthropometric parameters, such as weight, weight *Z* score, BMI, BMI *Z* score, SMM, FFM, and TBW differed significantly (see Section 3 and Table 1). As BC parameters depend on age and BMI, a propensity score matching on a 1:1 basis of healthy children to patients were performed. Pairs of HC and patients with IBD matched for age, sex, and BMI were formed. The maximum inter-individual difference allowed within a pair was <1 year in age and 1 kg/m^2^ in BMI.

Age, sex, and BMI normalized reference values for SMM were generated by the LMS method, which characterizes the distribution of a variable by its median (M), the coefficient of variation (S, i.e., the ratio of the SD and mean), and skewness (L) required to transform the data to normality (35). To evaluate this, a maximum-likelihood curve-fitting algorithm to the original data plotted over the independent variable was used. One set of tables was created using BMI *Z* scores as independent variable for calculating SMM *Z* score values in each percentile group. L, M, and S can be used to create percentiles (C_α_) according to Equation (1):

(1) $$C_{\alpha }(t)=M(t)\times [1+L(t)\times S(t)\times z_{\alpha }{]}^{1/L(t)}$$

where M(t), L(t), and S(t) or C_α_(t) indicate the corresponding values of each parameter at a given age, sex, and BMI. z_α_ is the normal equivalent deviate corresponding with the centile (e.g., *α* = 50, z*_α_* = 0; *α* = 75, z*_α_* = 0.674; *α* = 90, z*_α_* = 1.282; *α* = 95, z*_α_* = 1.645; and *α* = 97, z*_α_* = 1.881). Equation (1) can be rearranged to convert an individual child's SMM *Z* score value to the following standard deviation score (SDS):

(2) $$Z,\;i.e.,\;\mathrm{SDS}=\left\{{\left[Y/M(t)\right]}^{L(t)}-1\right\}/\left[L(t)\times S(t)\right]$$

where Y is the individual parameter of a child (SMM), and L(t), M(t), and S(t) are the specific values of L, M, and S interpolated for the BMI *Z* score of the same child (22). During the analysis, less than 5% (*p* < 0.05) probability was considered to give a statistically significant chance that the difference found is not a coincidence.

### 2.4 Ethics

Ethical permission for the measurement of IBD children was provided by the Semmelweis University's Institutional Committee for Research Ethics (SE TUKEB No: 215/2016). Healthy children of HC group were examined at local primary and secondary schools between 2007 and 2012 (OTKA 071730 approval, National Scientific Research Fund, participating authors: GR and OC). Parental informed consent was a prerequisite for inclusion to the study both for HC and IBD. The study was performed in accordance with the Declaration of Helsinki.

## 3 Results

### 3.1 Baseline characteristics

On the whole, 57 patients with IBD (31 with Crohn's disease, 26 ulcerative colitis) and 307 healthy children and adolescents were enrolled in this study. Patients with IBD were at the same age as the entire healthy cohort. Those in the IBD group had the same height and height *Z* score as compared to HC, and had lower weight and BMI than that of non-adjusted healthy controls. BMI *Z* scores of patients were lower, but calculated BMI showed nearly the same mean values both in healthy and IBD population. SMM, FFM, and TBW were lower in IBD group; meanwhile, BFM did not differ significantly between groups. Baseline characteristics of the normal population and patients with IBD are summarized in Table 1 and Supplementary Table S2.

Descriptive statistics of the healthy population according to age groups are shown in Supplementary Table S1. In the HC group, SMM was significantly higher in males than females (26.7 ± 7.5 kg, *n* = 143 vs. 22.3 ± 4.0 kg, *n* = 164, *p* < 0.0001); this difference was not observed among patients with IBD (22.9 ± 7.8 kg, *n* = 32 vs. 21.5 ± 9.1 kg, *n* = 25, *p* = 0.54).

From the 31 patients with CD (mean age: 14.1 ± 2.6 years, 19 males), 20 (65%) were newly diagnosed and 11 (35%) were starting anti-TNF therapy due to refractoriness to conservative therapy according to the guidelines at the time of the BC assessment (24, 25). Of the 26 patients with UC (mean age: 14.2 ± 2.9 years, 13 males), 14 (54%) were newly diagnosed and 12 (46%) started anti-TNF treatment. Clinical characteristics, anthropometric, and laboratory parameters of patients are summarized in Table 1 and Supplementary Table S2.

### 3.2 BMI-based *Z* score calculation

To analyze the relationship between BC, demographic, and anthropometric data, we developed a Pearson correlation matrix. SMM, FFM, TBW, and BFM were associated significantly with age (*r* = 0.65, 0.64, 0.66, and 0.21, respectively; *p* < 0.05), sex (*r* = −0.35, −0.32, −0.35, and 0.23, respectively; *p* < 0.05), weight (*r* = 0.9, 0.87, 0.91, and 0.49, respectively; *p* < 0.05), height (*r* = 0.87, 0.87, 0.89, and 0.17, respectively; *p* < 0.05), and BMI (*r* = 0.62, 0.58, 0.61, and 0.66, respectively; *p* < 0.05). Next, a multivariate linear regression model was adjusted to age, sex, height, weight, and BMI, and it showed that the SMM was significantly associated with all parameters. Thus, a stepwise ridged analysis was calculated and found age, sex, and BMI as significant determinants (β = 0.45, −0.31, and 0.38, respectively; *p* < 0.05) of SMM.

**Figure 1:**
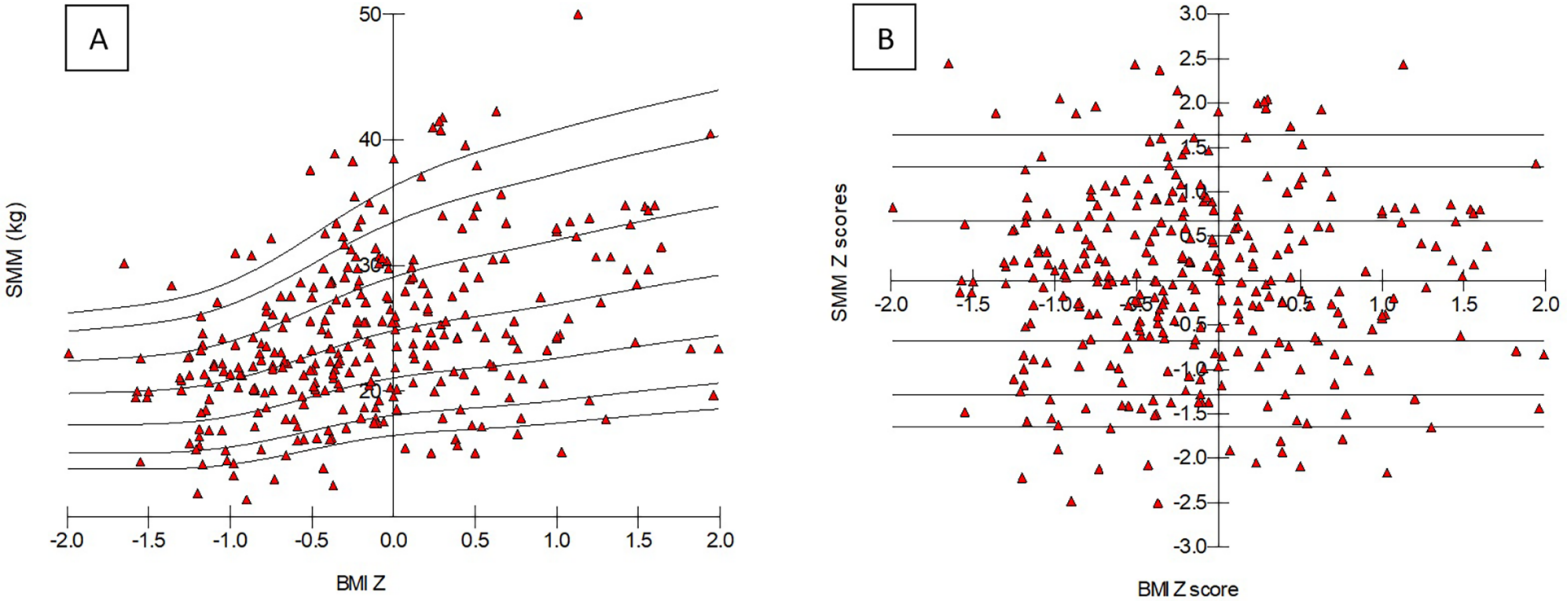
BMI *Z* scores–based percentile curve of skeletal muscle mass parameters **(A)** and *Z* score values **(B)** in healthy controls. Triangles show each child from the control population. SMM, skeletal muscle mass; BMI, body mass index.

Since patients with IBD had lower BMI and BC values as compared to HC and all were influenced by age and sex, a propensity score matched control population was created (Table 2). Surprisingly SMM did not show any significant difference between IBD and adjusted HC. Thus, to show whether children with IBD have true low muscle mass featured by SMM, without using propensity score matching, SMM *Z* score calculation was implemented as per BMI percentiles by using the LMS method having been detailed in the Section 2.3. Values of SMM were determined, and percentile boundaries were calculated and plotted by normalizing to age, sex, and BMI. BMI *Z* score–based LMS calculation of SMM was carried out, and percentile curves were plotted accordingly (Table 3 and Figure 1A). BMI *Z* score did not show significant association with SMM *Z* score (Figure 1B). An individual child's SMM *Z* score value was calculated according to the equation above in the Section 2.3.

**Table 2:** Comparison of anthropometric and body composition parameters between patients and controls after adjusting for age, sex, and BMI.

Variables	Adjusted controls		Patients	
	Mean	95% CI	Mean	95% CI
*n*	55		57	
Age (years)	14.4	13.8–15.1	14.2	13.5–15.0
Sex [males (%)]	32 (58)		32 (56)	
Height (cm)	165.5	162.1 to 168.9	161.9	158.1 to 165.8
Height *Z* score	0.5	0.3 to 0.8	0.3	0.0 to 0.6
Weight (kg)	49.7	46.6 to 52.7	46.9	43.7 to 50.1
Weight *Z* score	−0.4	−0.6 to −0.1	−0.5	−0.8 to −0.2
BMI (kg/m^2^)	17.9	17.3 to 18.6	17.6	19.6 to 20.4
BMI *Z* score	−0.6	−0.9 to 0.5	−0.7	[−0.9 to −0.5]
SMM (kg)	23.4	21.7 to 25.1	22.3	20.1 to 24.6
FFM (kg)	42.5	39.7 to 45.3	37.9*	35.4 to 40.6
TBW (L)	31.1	29.1 to 33.2	27.9*	25.9 to 29.8
BFM (kg)	7.2	5.9 to 8.4	8.6	7.2 to 10.0

BMI, body mass index; TBW, total body water; FFM, fat-free mass; BFM, body fat mass; SMM, skeletal muscle mass.

* *p* < 0.05.

**Table 3:** LMS values and specific percentile limit for SMM according to BMI *Z* scores.

Variable	L	M	S	5th	10th	25th	50th	75th	90th	95th
BMI *Z* score										
−1.99	0.79	19.84	0.19	13.85	15.13	17.33	19.84	22.42	24.79	26.24
−1	0.56	20.90	0.21	14.19	15.58	18.01	20.90	23.97	26.90	28.72
0	0.18	24.82	0.24	16.49	18.10	21.06	24.82	29.10	33.46	36.32
1	0.11	26.98	0.26	17.47	19.27	22.63	26.98	32.05	37.31	40.82
1.99	0.24	29.23	0.26	18.60	20.64	24.43	29.23	34.72	40.30	43.95

L, skeweness; M, median; S, coefficient of variation, SMM, skeletal muscle mass; BMI, body mass index.

### 3.3 Application of SMM Z score in children with IBD

Mean SMM *Z* score for patients with IBD were −0.03 (95% CI: −0.5 to 0.5). Further SMM values for patients with IBD are shown in Supplementary Table S1. The difference between CD and UC patients’ SMM *Z* score did not reach the level of significance (CD: 0.1; 95% CI −0.6 to 0.9 vs. UC: −0.18; 95% CI −0.7 to −0.4). Further data are available in Supplementary Table S2.

## 4 Discussion

Our cross-sectional study suggests that BC measurements in children with IBD are not conclusive in the diagnosis of sarcopenia without taking into consideration its association with age, sex, and BMI. Our study was the first to show that the degree of muscle loss (SMM) depends on age, sex, and BMI. Moreover, other BC parameters (FFM, TBW, and BFM) are also related to sex and BMI. Using unadjusted SMM values seems to overestimate the amount of muscle loss during IBD disease course. The use of SMM *Z* score (SMM normalized for age and BMI) seems to be needed to detect the real loss of muscle mass, and it contributes to attain a reliable estimation method of sarcopenia in children with IBD by showing the pathophysiological deviation from normal muscle mass of BMI *Z* score adjusted HCs. Accordingly, normalization of the BC parameters applied to characterize sarcopenia in childhood is desirable due to the evidence that the alteration of muscle mass is effected by the growth process, similar to other parameters used in clinical practice (for instance, blood pressure or pulse wave velocity) (22). Our results provide a simple tool to estimate the SMM percentile according to the calculated BMI *Z* score. It could provide an easy-to-measure option to calculate SMM *Z* score and to assess the net SMM loss compared to healthy counterparts with the same age and body dimensions.

We aimed to find the main factors determining SMM and to assess the loss of SMM in patients with IBD. Our analysis showed that age, sex, and BMI were the main determinants of SMM. Therefore, based on the data of the healthy cohort, BMI *Z* score–based SMM *Z* scores were required to be calculated and applied in children with IBD. We found that patients with IBD had decreased weight and BMI *Z* scores compared to the healthy cohort. However, they did not have significantly diminished SMM using our BMI-based SMM *Z* scores.

It is known from earlier studies, that not just genetics but environmental factors also influence BC, and BC differs among ethnicities, races, body shapes, and sizes. Therefore, to evaluate BC parameters in pediatric patients, population-specific BC reference curves are needed (36). Previously, age and sex reference curves were created for fat mass and FFM in different countries (e.g., USA, Canada, Korea, Netherlands). However, in these studies, the determinants of BC parameters were not analyzed (14, 17, 37).

The idea of relating FFM to height comes from Forbes, who demonstrated in 1972 that FFM is related to the cube of height (38). Next, VanItallie et al. split BMI into two parts: lean mass index (LMI; lean mass/height^2^) and fat mass index (FMI; fat mass/height^2^) (39). They suggested that FFM was independent of body size. In 2012, Wang et al. followed this concept and examined the influence of sex and population ancestry on the association of FFM and height in children and adults (40). However, they did not analyze other factors determining FFM (only height). Accepting this approach, population-specific FFM index and LMI reference curves were developed for children (17, 37). In our IBD population, FFM was diminished and TBW content was lower after adjusting for age and sex, which supports the potential direct pathophysiological effect of IBD as a chronic wasting disease in this patient population. Our result suggests that TBW and FFM parameters seem to be applicable for estimating volume overload and fat loss without normalization for BMI and can be used as surrogate markers of BC in patients with IBD.

Although we found a significant association between height and SMM, after the ridge regression analysis, age, sex, and BMI remained the main determinants of SMM. This finding is in line with our knowledge about BIA and the approach of some previous studies. BMI has an independent effect on the basic parameters of BIA, such as reactance and resistance (41). These are the basic parameters of BIA, from which BC parameters such as SMM are computed (41). Interestingly, phase angle, another raw parameter of BIA that can be used for assessing malnutrition and clinical prognosis, is also associated with BMI in children but not adults (42). Furthermore, aiming to assess the metabolic risk, McCarthy et al. also evaluated muscle-to-fat ratio according to BMI, age, and sex in children (16). From this point of view, our findings are supported by earlier studies. However, to the best of our knowledge, this is the first time that sex, age, and BMI-based SMM *Z* scores were developed.

In this study TBW, BFM, and FFM showed association with sex and BMI, and SMM correlated with age, sex, and BMI. After propensity score matching, SMM and BFM did not differ from the HCs. The remaining decrease in TBW and FFM supports the fact that the IBD group might suffer from a specific type of malnutrition and raises the suspicion that reduced unadjusted SMM of the children with IBD is rather a physiological deviation than could be explained by direct wasting due to the presence of chronic illness. Thus, in malnourished, unnurtured children, direct measurement of SMM does not seem to be eligible for measuring the rate of muscle wasting adequately due to its association with aging and age-related changes of body size. Therefore, SMM normalization according to BMI percentile groups is needed to precisely monitor the muscle loss caused by chronic wasting diseases that could represent real SMM in children with chronic wasting diseases.

It is not straightforward to compare this result with other studies. Previous studies often used FFM or lean mass to characterize muscle mass (43). Few studies investigated muscle mass, calculating sex-, age-, and height-specific *Z* scores (based on the fact that growth retardation is common in pediatric patients with IBD). These studies reported lower muscle mass than controls (18, 19). Interestingly, Werkstetter et al. calculated muscle-specific values with and without correction for height and described less decreased values after correction (44). We consider that in our IBD cohort, the lower “raw” SMM values compared to the unadjusted control group are likely due to smaller body size or growth retardation and do not reflect the accurate muscle mass.

Our study had several limitations. As SMM has not been identified as a biomarker of sarcopenia, and as the definition of pediatric sarcopenia is controversial, our study could only provide a crude approach to assess probable muscle loss as a potential measure of significant sarcopenia in the clinical scenario of malnutrition in patients with IBD. Our study was a cross-sectional single-center report. Thus, the predictive role of the SMM *Z* score could not be studied thus far. Also, the study involved only Caucasian children; therefore, our results cannot be generalized to children of other ethnicities. No potential validation option has been included in the study protocol that could objectively support the fact that SMM is a direct measure of sarcopenia and represents actual muscle mass loss in children with IBD. The frame of this manuscript could not provide space for analyzing all BC parameters in detail. BC may be influenced by diet, disease activity, and disease location; however, due to the low number of patients, we were not able to analyze this question in our study.

In conclusion, malnutrition in IBD children can be monitored with BIA measurements. However, due to age, sex, and body size dependence of certain BC parameters, their use as a diagnostic tool should be treated with caution. TBW and FFM parameters seem to be applicable for estimating volume overload and fat loss without normalization for BMI. SMM adjustment for BMI *Z* score proved to be desirable though due to its notable association with growing and aging process. BMI-based SMM *Z* score may serve as an objective estimator of muscle loss in children with wasting diseases, and it helps avoid overestimation of sarcopenia. We propose implementing this data-driven approach for estimating muscle mass loss in pediatric care units specializing in the treatment of wasting diseases, where BIA devices are utilized for BC assessment. This method offers a standardized and evidence-based means of evaluating muscle mass depletion in these clinical setting. The determination of the cut-off values for BC parameters, the proof of the predictive role of SMM *Z* score as novel practical approach to monitor muscle loss, and their eligibility for precise characterization of sarcopenia in children suffering from chronic wasting diseases remain to be investigated further.

## Data Availability

The raw data supporting the conclusions of this article will be made available by the authors, without undue reservation.
