# Supplementary material for: Novel approach to assess sarcopenia in children with inflammatory bowel disease
Source: Front Pediatr. 2024 Nov 19;12:1204639. doi: 10.3389/fped.2024.1204639 (PMC11611584; doi:10.3389/fped.2024.1204639)
Supplement: Supplementary file 1 [file Table1.docx]

Supplementary Material

Supplement Table 1.: Healthy controls according to age groups

Supplement Table 2.: Clinical, anthropometric and laboratory data of patients with inflammatory bowel disea

| **Supplement Table 1.  Healthy controls according to age groups** | | | | | | | | | | | | | | | | | | |
| --- | --- | --- | --- | --- | --- | --- | --- | --- | --- | --- | --- | --- | --- | --- | --- | --- | --- | --- |
| **Variables / Age groups (min to max)** | 9.98 to 10.89 | | 11 to11.92 | | 11.98 to 12.95 | | 12.95 to 13.94 | | 14.02 to 14.92 | | 14.99 to 15.94 | | 15.95 to 16.90 | | 16.97 to 17.90 | | 18 to 19.39 | |
| n | 20 | | 34 | | 31 | | 54 | | 38 | | 54 | | 46 | | 21 | | 9 | |
|  | mean | CI (95%) | mean | CI (95%) | mean | CI (95%) | mean | CI (95%) | mean | CI (95%) | mean | CI (95%) | mean | CI (95%) | mean | CI (95%) | mean | CI (95%) |
| Age (years) | 10.5 | (10.3-10.6) | 11.5 | (11.4-11.6) | 12.49 | (12.381-12.607) | 13.40 | (13.32-13.48) | 14.55 | (14.47-14.63) | 15.45 | (15.37-15.53) | 16.38 | (16.297-16.467) | 17.35 | (17.201-17.489) | 18.53 | (18.12-18.94) |
| Sex (males (%)) | 9 (45) | | 18 (52.9) | | 11 (35.5) | | 28 (52.8) | | 18 (47.4) | | 30 (55.5) | | 19 (41.3) | | 5 (23.8) | | 5 (55.5) | |
| Height (cm) | 143.8 | (141.4-146.2) | 151.1 | (148.2-154.0) | 157.57 | (154.69-160.44) | 163.09 | (160.97-165.22) | 169.59 | (167.05-172.14) | 170.48 | (168.5-172.463) | 171.87 | (169.34-174.40) | 170.92 | (166.31-175.54) | 174.67 | (168.58-180.76) |
| Height Z score | 0.3 | (-0.1-0.7) | 0.4 | (0.0-0.8) | 0.44 | (0.02-0.86) | 0.51 | (0.24-0.79) | 0.64 | (0.310.97) | 0.34 | (0.075-0.595) | 0.44 | (0.13-0.75) | 0.35 | (-0.20-0.89) | 0.69 | (0.19-1.2) |
| Weight (kg) | 36.4 | (33.4-39.3) | 41.7 | (38.9-44.6) | 44.79 | (41.74-47.84) | 49.60 | (47.29-51.92) | 56.78 | (53.70-59.86) | 60.42 | (57.544-23071) | 62.32 | (59.39-65.25) | 60.81 | (55.74-65.87) | 63.20 | (56.37-70.03) |
| Weight Z score | 0.1 | (-0.3-0.4) | 0.1 | (-0.2-0.3) | -0.11 | (-0.41-0.18) | -0.10 | (-0.304-0.11) | 0.06 | (-0.21-0.34) | 0.14 | (-0.133-0.413) | 0.14 | (-0.10-0.38) | -0.07 | (-0.48-0.35) | 0.30 | (-0.39-0.99) |
| BMI (kg/m2) | 17.5 | (16.4-18.7) | 18.2 | (17.3-19.0) | 17.94 | (17.09-18.79) | 18.58 | (17.90-19.26) | 19.68 | (18.87-20.50) | 20.69 | (19.952-21.426) | 21.02 | (20.34-21.70) | 20.72 | (19.52-21.92) | 20.61 | (19.58-21.65) |
| BMI Z score | -0.1 | (-0.4-0.3) | -0.1 | (-0.3-0.2) | -0.35 | (-0.59--0.11) | -0.35 | (-0.55--0.15) | -0.22 | (-0.46-0.03) | -0.02 | (-0.246-0.21) | -0.06 | (-0.28-0.16) | -0.25 | (-0.63-0.14) | 0.24 | (-0.39-0.88) |
| TBW (l) | 21.3 | (20.2-22.5) | 24.50 | (23.1-25.9) | 27.07 | (25.63-28.52) | 30.60 | (29.35-31.85) | 35.04 | (33.21-36.87) | 36.29 | (34.603-37.978) | 37.14 | (34.89-39.39) | 35.81 | (32.27-39.36) | 39.40 | (33.24-45.56) |
| FFM (kg) | 29.2 | (27.7-30.9) | 33.70 | (31.8-35.6) | 36.95 | (34.98-38.91) | 41.84 | (40.14-43.54) | 47.87 | (45.38-50.35) | 48.63 | (45.773-51.489) | 50.86 | (47.76-53.95) | 49.05 | (44.21-53.89) | 53.79 | (45.47-62.11) |
| BFM (kg) | 7.3 | (5.3-9.2) | 8.60 | (6.8-10.4) | 7.84 | (6.21-9.46) | 8.58 | (6.54-10.63) | 8.91 | (7.15-10.68) | 10.83 | (8.967-12.685) | 11.31 | (9.92-12.71) | 11.76 | (9.52-14.00) | 9.40 | (6.50-12.30) |
| SMM (kg) | 15.8 | (14.9-16.7) | 18.30 | (17.3-19.4) | 20.04 | (18.80-21.27) | 23.16 | (22.14-24.17) | 26.68 | (25.20-28.16) | 27.82 | (26.36-29.284) | 28.57 | (26.65-30.49) | 27.40 | (24.49-30.31) | 30.37 | (25.31-35.42) |
| Data are shown as mean and 95% CI. Abbreviations: CI- confidence interval; BMI - body mass index; TBW - total body water; FFM - fat free mass; BFM - body fat mass; SMM - smooth muscle mass *p<0.05 | | | | | | | | | | | | | | | | | | |

| **Supplement Table 2.  Clinical, anthropometric and laboratory data of patients with inflammatory bowel disease** | | | | | | |
| --- | --- | --- | --- | --- | --- | --- |
| **Variables** | **IBD** | | **Crohn's disease** | | **Ulcerative colitis** | |
|  | mean | CI (95%) | mean | CI (95%) | mean | CI (95%) |
| Number of patients | 57 | | 31 | | 26 | |
| Age in years | 14.17 | (13.45 - 14.89) | 14.11 | (13.18 - 15.05) | 14.24 | (13.06. - 15.43) |
| Sex (male, n (%)) | 32 (56) | | 19 (61) | | 13 (50) | |
| Anthropometric and body composition parameters | | | | | | |
| Height (cm) | 161.9 | (158.1-165.8) | 160 | (156.1 - 165.3) | 163.5 | (156.8-170.1) |
| Height Z score | 0.28 | (-0.02 - 0.58) | 0.14 | (-0.26 - 0.54) | 0.44 | (-0.02-0.91) |
| Weight (kg) | 46.91 | (43.71-50.1) | 45.63 | (41.59 - 49.67) | 48.43 | (43.1-53.77) |
| Weight Z score | -0.5 | (-0.75-(-0.24)) | -0.55 | (-0.94 - (-0.16)) | -0.44 | (-0.79-(-0.08)) |
| BMI (kg/m2) | 17.63 | (16.89-18.36) | 17.51 | (16.47 - 18.56) | 17.76 | (16.66-18.86) |
| BMI Z score | -0.71 | (-0.94-(-0.47)) | -0.69 | (-1.06 - (-0.33) | -0.72 | (-1.02-(-0.42)) |
| SMM (kg) | 22.34 | (20.12 - 24.57) | 23.1 | (19.48 - 26.73) | 21.43 | (18.92-23.94) |
| SMM Z score | -0.03 | (-0.5 - 0.5) | 0.1 | (-0.6 - 0.9) | -0.18 | (-0.7 - 0.4) |
| FFM (kg) | 37.99 | (35.39 - 40.58) | 36.6 | (33.45 - 39.86) | 39.57 | (35.19-43.95) |
| FFM Z score | -0.3 | (-0.6 - (-0.0)) | -0.4 | (-0.9 - (-0.09)) | -0.14 | (-0.6 - 0.4) |
| TBW (l) | 27.86 | (25.93 - 29.78) | 26.87 | (24.5 - 29.24) | 29.03 | (25.77-32.29) |
| TBW Z score | -0.3 | (-0.6-(-0.0)) | -0.4 | (-0.9 - (-0.08)) | -0.13 | (-0.6-0.4) |
| BFM (kg) | 8.559 | (7.151 - 9.96) | 8.3 | (6.45 - 10.15) | 8.86 | (6.56-11.16) |
| BFM Z score | 0.4 | (0.06 - 0.7) | 0.3 | (-0.2 - (-0.08)) | 0.5 | (0.05 - 1.04) |
| Disease activity indices | | | | | | |
| PCDAI/PUCAI (CI) |  |  | 22.3 | (16.83 - 27.69) | 35.58 | (27.18 - 43.98) |
| PCDAI ≤10 n (%) |  |  | 8 (25) | |  |  |
| PCDAI 11-29 n (%) |  |  | 15 (48) | |  |  |
| PCDAI 30-39 n (%) |  |  | 4 (12) | |  |  |
| PCDAI ≥40 n (%) |  |  | 4 (12) | |  |  |
| PUCAI < 10 n (%) |  |  |  |  | 3 (11) | |
| PUCAI 10-34 n (%) |  |  |  |  | 8 (30) | |
| PUCAI 35-64 n (%) |  |  |  |  | 12 (46) | |
| PUCAI 65< n (%) |  |  |  |  | 3 (11) | |
| Laboratory parameters | | | | | | |
| CRP (mg/l)** | 20.43 | (12.54 - 28.32) | 30.4 | (18.38 - 42.35) | 8.1 | (0.19 - 16.02) |
| Haemoglobin (g/dL)* | 120.1 | (115.9 - 124.4) | 116.2 | (110.9 - 121.5) | 125 | (118.2 - 131.8) |
| Thrombocytes (G/L)** | 414.4 | (368.5 - 460.2) | 472 | (416.6 -527.3) | 343 | (272.6 - 413.4) |
| Albumin (g/L)** | 41.4 | (39.68 - 42.39) | 39.4 | (37.53 - 41.24) | 43.08 | (41.29 - 44.87) |
| Extent / Location of the disease | | | | | | |
| L1 (Ileal) n (%) |  |  | 9 (29) | |  |  |
| L2 (Colonic) n (%) |  |  | 7 (22.5) | |  |  |
| L3 (Ileocolonic) n (%) |  |  | 15 (48.4) | |  |  |
| L4 (Upper gastrointestinal tract) n (%) |  |  | 16 (51.6) | |  |  |
| E1 (Proctitis) n (%) |  |  |  |  | 1 (3.8) | |
| E2 (Left-sided) n (%) |  |  |  |  | 12 (46) | |
| E3 (Extensive) n (%) |  |  |  |  | 2 (7.7) | |
| E4 (Pancolitis) n (%) |  |  |  |  | 11 (42.3) | |
| Disease Behaviour n (%) |  |  |  |  |  |  |
| B1 Non-stricturing. non-penetrating n (%) |  |  | 25 (80) | |  |  |
| B2 Stricturing n (%) |  |  | 5 (16) | |  |  |
| B3 Penetrating n (%) |  |  | 1 (3.2) | |  |  |
| S0 n (%) |  |  |  |  | 16 (61) | |
| S1 n (%) |  |  |  |  | 10 (38) | |
| Medical treatment % (n) | | | | | | |
| 5-ASA n (%) | 42 (73) | | 18 (58) | | 23 (88) | |
| AZA n (%) | 37 (65) | | 24 (77) | | 12 (46) | |
| MTX n (%) | 1 (0.02) | | 0 | | 1 (0.04) | |
| Antibiotics n (%) | 10 (17) | | 8 (25) | | 2 (0.07) | |
| Systemic corticoid n (%) | 15 (27) | | 4 (13) | | 10 (38) | |
| Topical steroid n (%) | 6 (10.5) | | 4 (13) | | 1 (0.04) | |
| Exclusive enteral nutrition n (%) | 16 (28) | | 16 (51) | | 0 | |
| Adalimumab n (%) | 18 (31.5) | | 8 (25.8) | | 9 (34) | |
| Infliximab n (%) | 5 (8) | | 3 (9) | | 2 (0.7) | |
| Data are shown as mean and 95% CI. Abbreviations: CI- confidence interval; BMI - body mass index; TBW - total body water; FFM - fat free mass; BFM - body fat mass; SMM - smooth muscle mass. PCDAI:paediatric Crohn's disease activity index. PUCAI: paediatric ulcerative colitis activity index. S0: clinical remission. S1: mild UC. 5-ASA: 5-aminosalicylic acid. AZA: azathioprine. MTX: methotrexate * <0.05; ** < 0.005 | | | | | | |
